# A case of bilateral abdominoscrotal hydroceles without communication with the peritoneum

**DOI:** 10.4103/0970-1591.60461

**Published:** 2010

**Authors:** Eiji Hisamatsu, Shizuko Takagi, Masashi Nomi, Yoshifumi Sugita

**Affiliations:** Department of Urology, Kobe Children's Hospital, Kobe, Japan

**Keywords:** Abdominoscrotal hydrocele, communication, peritoneum

## Abstract

Abdominoscrotal hydrocele (ASH) is an uncommon entity. Although various theories on the development of ASH have been proposed, its etiology is still unclear. According to several etiological theories, it is necessary that ASH have communication with the peritoneum. We present a case of bilateral ASH that had no communication with the peritoneum.

## INTRODUCTION

Abdominoscrotal hydrocele (ASH) is an unusual condition in which a scrotal hydrocele has a dumbbell-shaped extension through the deep ring into the abdomen. Although various theories about the development of ASH have been proposed, they do not explain why ASH is rare while communicating scrotal hydroceles are frequent in children. Herein we report a case of bilateral ASH that had no communication with the peritoneum.

## CASE REPORT

**Figure 1:**
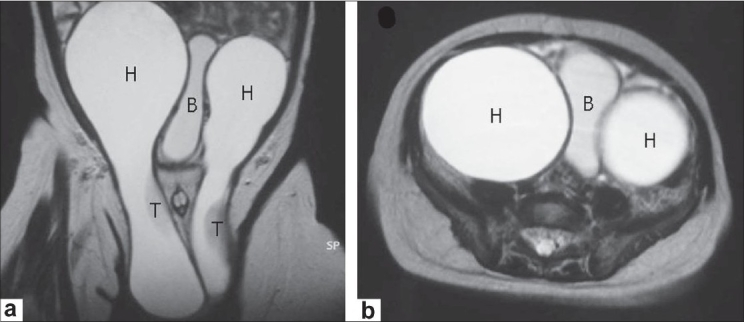
T2-weighted magnetic resonance images from the coronal; (a) axial; (b) views. The bilateral massive hydroceles extended into the abdomen through the inguinal canal. Hydrocele; H, Bladder; B, Testis; T

**Figure 2:**
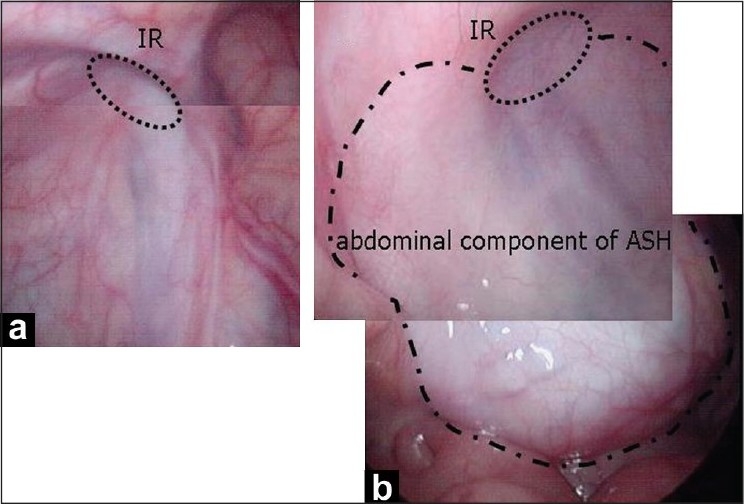
Laparoscopic findings in the left; (a) right; (b) sides. No patent processus vaginalis was found on either side. The abdominal cavity was compressed extraperitoneally by the abdominal component of the right abdominoscrotal hydrocele. IR shows the internal inguinal ring

A 3-month-old boy was referred with a history of bilateral scrotal hydroceles since birth. On physical examination, large inguinoscrotal hydroceles were noted on both sides. After a 3-month period of observation, ultrasonography (US) showed extension of the bilateral hydroceles through the inguinal canal into the abdomen. Magnetic resonance imaging (MRI) confirmed the US findings [Figure 1]. Surgical repair was scheduled for 2 months later. Laparoscopy was performed through an infraumbilical incision before hydrocelectomies. No patent processus vaginalis was found on either side. The abdominal cavity was compressed extraperitoneally by the abdominal component of the right ASH [Figure 2]. Subsequently, bilateral hydrocelectomies were performed through bilateral inguinal incisions. The procedure was difficult because of adherence of the hydroceles to the spermatic cord. No obvious communication between the peritoneum and the hydrocele was found on either side. Only thin connective tissues were observed. The abdominal portion of the ASH was completely resected, whereas the inguinoscrotal portion was reduced with particular attention to the spermatic vessels and vas deferens. Bilateral high ligations of the peritoneum at the internal inguinal ring (IR) were added to prevent inguinal hernias because the IRs were enlarged by the hydroceles. The patient has been doing well postoperatively without evidence of recurrence.

## DISCUSSION

ASH is an uncommon entity. There are different theories to explain the development of this condition. According to several theories, the peritoneal communication is necessary, and the hydrocele has a one-way valve mechanism.[1] The valve mechanism does not adequately account for the development of ASH in our case because there was no obvious communication between the peritoneum and the hydrocele according to laparoscopic and surgical findings. We suggest another mechanism of the development of ASH. ASHs without communication may result from increased production or decreased resorption of serous fluid such as non-communicating hydroceles in adults.

Early surgical correction of ASH had been recommended because spontaneous resolution had not been documented. However, surgery is often difficult because of adherence of the hydrocele to the cord structures such as that seen in our case.[2] The scrotal approach reported by Belman[3] can be used as an alternative method if no communication is identified between the hydrocele and the peritoneum by laparoscopy. Recently, spontaneous resolution of ASH has been documented in several reports.[4,5] Watchful waiting is an alternative option, although it may be difficult to persuade parents concerned about a large scrotal swelling.

## CONCLUSIONS

ASHs that have no communication with the peritoneum may result from the same mechanism as non-communicating hydroceles in adults. Particular attention should be paid to the spermatic vessels and vas deferens during transinguinal excision because the sac might be adherent to the cord structures.
